# 

**Published:** 1909-05-15

**Authors:** 


### THE GRADUATES' MEDICAL SCHOOLS.

DIARY FOR THE WEEK, MAY 17 TO MAY 22.

### THE THROAT HOSPITAL, Golden Square, W.

**At 5.30 p.m.**
**May 17,** Mr. Parker, **Chronic Inflammatory Affections of the Middle Ear.**
**May 20,** Mr. Parker, **Otosclerosis.**

### THE POST-GRADUATE COLLEGE, West London Hospital, Hammersmith, W.

**At 10 a.m.**
**May 17 and 20,** Surgical Registrar, **Demonstration.**
**May 21,** Medical Registrar, **Demonstration.**
**At 12 noon.**
**May 17,** Dr. Bernstein, **Pathological Demonstration.**
**At 12.15 p.m.**
**May 19,** Dr. Pritchard, **Practical Medicine.**
**At 5 p.m.**
**May 18,** Dr. Moullin, **Gynæcology.**
**May 19,** Dr. Low, **Insects as Carriers of Disease in Tropical Medicine.**
**May 20,** Dr. Cole, **Melancholia and Allied Conditions.**
**May 21,** Mr. Harman, **How to Test the Vision of an Injured Eye—Workmen's Compensation.**

### MEDICAL GRADUATES' COLLEGE AND POLYCLINIC, 22 Chenies Street, W.C.

**At 5.15 p.m.**
**May 17,** Dr. Alexander Morison, **The Treatment of Cardiac Pains.**
**May 18,** Dr. G. E. Herman, **Some Points relating to Ectopic Pregnancy.**
**May 19,** Sir Thomas Oliver, **Some Unusual Features of Lead Poisoning.**
**May 20,** Dr. James Mackenzie, **A Demonstration of Graphic Methods for the Investigation of Cardio-Vascular Conditions.**

### NORTH-EAST LONDON POST-GRADUATE COLLEGE, Prince of Wales's General Hospital, Tottenham, N.

**At 4.30 p.m.**
**May 18,** Dr. G. N. Meachen, **Demonstration of Selected Skin Cases.**
**May 20,** Dr. A. G. Auld, **Pulmonary Emphysema.**

### LONDON SCHOOL OF CLINICAL MEDICINE, Seamen's Hospital, Greenwich, S.E.

**At 2.15 p.m.**
**May 21,** Dr. R. Bradford, **Hemiplegia.**

### THE FORTHCOMING SIXTEENTH INTERNATIONAL CONGRESS OF MEDICINE, BUDAPEST.

The detailed arrangements for the sixteenth International Congress of Medicine are now issued, with a list of the Committees and a programme of the communications promised to the sections. The meetings of the Congress will be held in the buildings of the former Polytechnic School Museum-korut 6/8, but the formal opening will take place in the Banqueting Hall of the Municipal Redoute at 11 A.M. on Sunday, August 29, 1909. The closing ceremony will occur at the same place on September 4, 1909, at 10 A.M. Six general sittings will be held during the Congress, at which addresses will be delivered by Professor Baccelli, of Rome; Dr. Bashford, of London; Professor Kutner, of Berlin; Dr. Laveran, of Paris; and Mr. J. Loeb, of Berkeley, U.S.A. A Journal will be issued daily, and there will be the usual secretarial offices and bureaux. Three new sub-sections have been formed—namely, orthopædics, professional hygiene, and school hygiene.

The General Secretary calls attention to the difficulty of finding adequate accommodation for intending visitors to Budapest unless they give timely notice to "The Central Travelling Ticket Office, iv. Vigado-tèr 1, Budapest, Hungary," and secure rooms by paying for them in advance. Certain concessions will be allowed by the railway companies to members attending the Congress. In Hungary the railways allow a reduction of 50 per cent. In France the management of the State railways and the East, North, and Orleans Companies also allow a 50-per-cent. reduction to members provided with the proper vouchers, whilst in England the G.E.R. grants a reduction to Congress members. Members of Congress who have paid their subscriptions will receive a circular about the end of May informing them of the method of claiming these reductions.

The Executive Committee offer six excursions for members after the Congress, each excursion being personally conducted and upon the ordinary inclusive terms, the first-class prices admitting of a single bedroom in hotels, whilst those who pay second class will be lodged in couples. The first excursion costs 150 crowns first class and 135 crowns second class (a crown is equivalent practically to a shilling). It includes a visit to Kolozsvar, the ancient capital of Transylvania, the Marosujvar salt-mines, and the Rév Pass. It leaves Budapest on September 4, and returns on September 7. Excursion No. 2 is to the High Tatra, Dobsina Ice Cavern, and Postyen Baths, leaving Budapest on September 4, returning September 9. Price 180 crowns first class, 150 crowns second class. Excursion 3 is to Lake Balaton, which is one of the largest and most beautiful in Europe. Members leave Budapest on September 4 and return on the 6th. Price 130 crowns first class, 110 crowns second class. Excursion No. 4 : The Lower Danube and Herkulesfürdö, leaving September 4, returning September 8. Fare, 185 crowns first class and 155 crowns second class. Excursion No. 5 : Constantinople, leaving Budapest September 4 and returning September 13. Price 475 crowns first class and 375 crowns second class. This excursion requires passports *viséd* at the Turkish and Roumanian Consulates. It allows five days in Constantinople. The excursionists having visited Constantinople may return *via* Trieste by putting into Athens and Corfu, the additional price being 425 crowns. Constantinople is left on September 11, and Trieste is reached on September 16. Excursion No. 6 : Bosnia, Herzegovina, Dalmatia, and the Hungarian Littoral, leaving Budapest on September 4 and returning on September 12. First class, 320 crowns; second class, 270 crowns.

The latest date of entry for any of the excursions is August 1, and in each case a booking fee has to be paid varying from 25 to 50 crowns, which is not repayable under any circumstances. Some of the excursions are necessarily limited in number, so that early application is desirable. So far as can be told at present, the Congress appears to be viewed with favour by the medical men of the United Kingdom, for it has secured a larger number of adherents than any of the Congresses since the one held in Paris. Communications should be addressed to the Honorary General Secretaries for Great Britain, Dr. Clive Rivière and Mr. D'Arcy Power, at 10A Chandos Street, Cavendish Square, London.

THE HOSPITAL May 15, 1909.
*Name* ..........................................
*Address* ..........................................
..........................................
This Coupon must accompany manuscript or contributions intended for THE HOSPITAL.

